# Proteomic Analysis of Bronchoalveolar Lavage Fluid: Effect of Acute Exposure to Diesel Exhaust Particles in Rats

**DOI:** 10.1289/ehp.9745

**Published:** 2007-02-05

**Authors:** John A. Lewis, K. Murali Krishna Rao, Vince Castranova, Val Vallyathan, William E. Dennis, Paul L. Knechtges

**Affiliations:** 1 U.S. Army Center for Environmental Health Research, Fort Detrick, Maryland, USA; 2 Pathology and Physiology Research Branch, Health Effects Laboratory Division, National Institute for Occupational Safety and Health, Morgantown, West Virginia, USA

**Keywords:** calprotectin, diesel, inflammation, macrophage, mass spectrometry, proteomics, SELDI

## Abstract

**Background:**

Inhalation of diesel exhaust particles (DEPs) is characterized by lung injury and inflammation, with significant increases in the numbers of polymorphonuclear leukocytes and alveolar macrophages. This influx of cellular infiltrates is associated with the activation of multiple genes, including cytokines and chemokines, and the production of reactive oxygen species.

**Objective:**

The pathogenesis of the lung injury is not fully understood, but alterations in the presence or abundance of a number of proteins in the lung have been observed. Our objective in this study was to further characterize these changes and to ask whether additional changes could be discerned using modern proteomic techniques.

**Methods:**

The present study investigates global alterations in the proteome of bronchoalveolar lavage fluid taken from rats 1, 7, or 30 days after exposure to 5, 35, or 50 mg/kg of animal weight of DEPs.

**Results:**

Analysis by surface-enhanced laser desorption/ionization–time of flight mass spectrometry identified two distinct peaks that appeared as an acute response postexposure at all doses in all animals. We identified these two peaks, with mass to charge ratios (*m/z*) of 9,100 and 10,100, as anaphylatoxin C3a and calgranulin A by additional mass spectral investigation using liquid chromatography coupled to mass spectrometry.

**Conclusions:**

With this approach, we found a number of inflammatory response proteins that may be associated with the early phases of inflammation in response to DEP exposure. Further studies are warranted to determine whether serum levels of these proteins could be markers of diesel exhaust exposure in workers.

In recent years low-molecular-weight serum protein profiling has become increasingly important in detecting early events in the disease process and predicting outcomes. The recent use of this technique to detect ovarian cancer provided a great impetus to this field (Petricoin et al. 2002). Both SELDI-TOF (surface-enhanced laser desorption/ionization–time of flight) and MALDI-TOF (matrix-assisted laser desorption/ionization–time of flight) mass spectrometry (Hutchens and Yip 1993; Karas and Hillenkamp 1988; Karas et al. 1987; Tang et al. 2004) are proving to be powerful tools for diagnosing disease states, particularly for early detection of cancer, through the analysis of proteomic patterns. When combined with bioinformatic tools, protein profiling has become an effective approach in screening for potential tumor markers (Rai et al. 2002).

Because bronchoalveolar lavage fluid (BALF) exhibits the cellular and biochemical alterations of inflammation and lung injury in response to various toxic agents, performance of a proteomic analysis of BALF to characterize the effects of diesel exhaust particles (DEPs) exposure is warranted. We previously designed a neural network program for the analysis of proteomic patterns in serum samples of humans exposed to various levels of DEPs (unpublished data). These studies showed the potential for proteomics to discriminate occupational exposures to various deleterious agents and prompted the validation study presented here.

DEP exposure induces the production of cytokines in lung epithelial cells *in vitro* (Bayram et al. 1998; Steerenberg et al. 1998) and in lung tissue *in vivo* (Saber et al. 2006). It also affects the lipopolysaccharide-induced production of cytokines (tumor necrosis factor-α and interleukin-1) in alveolar macrophages (AMs; Yang et al. 1997, 1999). We previously studied the expression of the mRNA levels for several of these cytokines and correlated these observations with the inflammatory response as assessed by measuring the influx of cells and protein into the bronchoalveolar space. In addition, cytokine levels were measured in BALF. The results showed that DEPs up-regulate several genes implicated in the inflammatory response, both at the message and protein levels, within 24 hr in cells obtained from BALF, representing the influx of both polymorphonuclear leukocytes (PMNs) and AMs (Rao et al. 2005).

In this study, we used newly available proteomic technologies to characterize the changes in protein concentrations caused by DEP exposure. We used a Ciphergen ProteinChip System and liquid chromatography coupled to mass spectrometry (LC/MS) to characterize the samples. In the Ciphergen system, protein samples are allowed to adsorb to spots on a fixed support with a specific surface chemistry. Unbound proteins are washed off the chip, and the remaining bound proteins are ionized with a laser, and their masses are characterized by time of flight mass spectrometry. For LC/MS, polypeptide mixtures are digested with trypsin; the peptides are bound to a chromatographic column, eluted with a continuous gradient of acetonitrile and ionized by electrospray directly into either a time of flight or ion-trap mass spectrometer. Using a weak cationic exchange ProteinChip, protein profiling was performed on BALF taken from rats at 1, 7, or 30 days after exposure to various concentrations of DEPs. This approach was complemented by global analysis using LC/MS to determine protein identity and to broadly screen for qualitative differences. We found DEP exposure–induced changes in the abundance of a number of proteins using a SELDI methodology. These and additional proteins identified by LC/MS are indicative of tissue damage and inflammation.

## Materials and Methods

### Animals

Research was conducted in compliance with the Animal Welfare Act (1966), and other federal statutes and regulations relating to animals and experiments involving animals and adheres to principles stated in the Guide for the Care and Use of Laboratory Animals (National Research Council 1996) in facilities fully accredited by the Association for the Assessment and Accreditation of Laboratory Animal Care, International. The animals were treated humanely and with regard for alleviation of suffering. The animals used in these experiments were specific pathogen-free male Sprague-Dawley rats [Hla:(SD)CVF; Hilltop Laboratories, Scottdale, PA], weighing 250–275 g (approximately 8 weeks old) at arrival. The rats were housed at the National Institute for Occupational Safety and Health animal facility, under temperature and humidity controlled conditions and a 12-hr light/dark cycle. The rats were monitored to be free of endogenous viral pathogens, parasites, mycoplasmas, *Helicobacter*, and CAR (cilia-associated respiratory) bacillus. Rats were acclimated for at least 5 days before use and were housed in ventilated cages, which were provided with HEPA-filtered air. Alpha-Dri virgin cellulose chips (Shepherd Speciality Papers, Watertown, TN) and hardwood Beta chips (NEPCO, Warrenburg, NY) were used as bedding. ProLab 3500 diet (Harlan Teklad, Madison, WI) and tap water were provided *ad libitum.*

### Reagents

DEPs were from a National Institute of Standards and Technology standardized heavy-duty diesel engine emission sample (no. 1650) with an average mass median diameter of 0.5 μm.

### Experimental design

Animals were exposed by intratracheal (IT) instillation with a single dose of either saline, or DEPs. Groups of animals (*n* = 4 per group), representing each treatment, were sacrificed at 1, 7, and 30 days after exposure to obtain BALF.

### Intratracheal instillation of DEPs

DEPs were suspended in endotoxin, Ca^2+^ and Mg^2+^ free phosphate-buffered saline (PBS; BioWhittaker, Walkersville, MD) and sonicated for 1 min. Rats were anesthetized with an intraperitoneal (ip) injection of 30–40 mg/kg body weight sodium methohexital (Brevital; Eli Lilly and Co., Indianapolis, IN) and were intratracheally instilled using a 20-ga, 4-inch ball-tipped animal feeding needle. Rats were given 5, 35, or 50 mg DEP/kg body weight or an equivalent volume of PBS. IT instillation has been shown to be a valid model to study pathological changes associated with airborne pollutants and is considered particularly useful in elucidating mechanisms of response (Henderson et al. 1995).

### BAL fluid

Rats were anesthetized with an overdose of sodium pentobarbital (100 mg/kg body weight) and exsanguinated. The trachea was cannulated, and the lungs were lavaged. BALF was obtained by a single lavage using cold Ca^2+^- and Mg^2+^-free PBS containing 5.5 mM d-glucose. The first lavage return of approximately 6 mL was centrifuged to sediment cells at 300 × *g*. The acellular supernatant was transferred to plastic tubes and stored at –80°C. Every effort was made to minimize protein degradation by avoiding multiple freeze–thaw cycles. Samples analyzed by SELDI-TOF were thawed only once. At times additional processing of samples, such as weak cation exchange (WCX)-extraction, required refreezing of aliquots.

### Proteomic patterns

Proteomic patterns were obtained using the WCX2 ProteinChip on the Ciphergen ProteinChip System (Ciphergen Biosystems, Inc., Fremont, CA). WCX2 chips were equilibrated with 2× binding buffer [50 mM ammonium acetate (NH_4_OAc) and 0.01% Triton X-100 at pH 6.0]. BALF samples were diluted 2-fold with binding buffer, and 200 μL was placed on a WCX2 chip in the bioprocessor and allowed to incubate for 1 hr. Chips were washed with binding buffer and water before drying and the addition of the sinapinic acid (SPA). Data collection was optimized for the mass to charge ratio (*m/z*) range of 3,000–50,000, with a detector sensitivity of 7, a laser intensity of 150 and a high *m/z* of 50,000. The data presented in the figures have been baseline subtracted using Ciphergen’s ProteinChip software with a window of 25 points for the option to smooth before fitting baseline and with the automatic option for parameters.

### Protein identification

Protein samples from a control rat and a rat 24 hr postexposure to the high dose were subjected to further analysis to identify the proteins associated with the peaks observed in the SELDI-TOF data. To mimic WCX2 chemistry, a solid-phase extraction (SPE) was performed with the BALF using a WCX resin, Biosepra CM Ceramic HyperD F (Pall Corp., East Hills, NY). The resin was equilibrated with 1× binding buffer. BALF was diluted with 2× binding buffer (1× final concentration) and mixed with the resin. The resin with bound BALF proteins was washed with 0.5 M NH_4_OAc to remove proteins with a low binding affinity, then proteins of interest were eluted with 2 M NH_4_OAc. Eluted proteins were separated using a NuPAGE 4–12% BisTris gel in an MES Running Buffer (Invitrogen Corp., Carlsbad, CA) and stained with SyproRuby (Bio-Rad Laboratories, Hercules, CA). Protein bands in the appropriate relative molecular weight range were manually excised, reduced with 5 mM dithiothreitol (DTT) and alkylated with 50 mM iodoacetamide (Bio-Rad Laboratories), digested with trypsin (Promega Corp., Madison, WI) and eluted by diffusion. After concentration by evaporation in a Speed Vac Concentrator (Eppendorf, Westbury, NY), samples were resuspended, and an aliquot of each elution was modified with imidazole to enhance ionization. Imidazole was part of the Lys Tag kit (Agilent Technologies, Palo Alto, CA) and used according to manufacturer’s recommendations. Both labeled and unlabeled samples were analyzed on an Agilent SL ion trap mass spectrometer connected to an Agilent 1100 nanoflow HPLC (Agilent Technologies).

As a secondary validation of identification, WCX-extracted proteins and whole BALF were analyzed by direct LC/MS (not gel based) on a Waters Q-Tof Premier quadrupole time-of-flight mass spectrometer (QTOF) in tandem with a nanoACQUITY ultra performance liquid chromatograph (UPLC) system (Waters Corp., Milford, MA). Before LC/MS, the samples were lyophilized and resuspended in 50 mM ammonium bicarbonate, 5 mM DTT and 0.05% RapiGest (Waters Corp.). After denaturation at 80°C, proteins were alkylated with 25 mM iodoacetamide and digested overnight with trypsin. The samples were diluted to a final RapiGest concentration of 0.025%. For the whole BALF, a tryptic digestion of the proteins was separated into eight fractions using an Agilent 1100 HPLC before analysis on the QTOF. In lieu of traditional tandem MS (MS/MS), QTOF data were collected using the Waters Protein Expression method. Three technical replicates were performed for each of the samples.

### Protein Expression method

This is a novel method of data acquisition and analysis designed by Waters to maximize information content gained from mass spectral analysis (Hughes et al. 2006; Silva et al. 2006). In this method, spectra are collected alternating between low- and high-collision energies; no selective mass filtering is performed. Therefore, fragmentation data are collected for every precursor ion and are not limited by the number of MS/MS scans that can be performed in a single run. Furthermore, the intensity of each precursor ion is collected across its entire peak, so quantitative data are maximized. The fragment ions in the high-energy scans are assigned to precursor ions based on elution profiles using computational methods. The collection of fragment ions is combined into a synthetic spectrum (termed MS^E^, where E signifies energy) that is used for database searches.

### LC parameters

Peptides extracted from gel spots were separated using an Agilent 1100 HPLC coupled to the Agilent SL ion trap mass spectrometer. The 8-μL injection volume was trapped on a 0.3 × 5–mm Zorbax SB C-18 column using 3% acetonitrile (MeCN) and 0.1% formic acid at a flow rate of 20 μL/min for 16 min. A 75-μm × 50-mm Zorbax SB C-18 column, 3.5-μm particle size (Agilent Technologies), was used for analytical separation with a flow rate of 300 nL/min. The gradient profile was 3% MeCN for 16 min, 10% MeCN at 23 min, 35% MeCN at 43 min, 80% MeCN at 48.5 min until 58.5 min, and 3% MeCN at 63 min until stopping at 67 min; 0.1% formic acid was used throughout.

Initial fractionation of whole BALF peptides was performed using a combination of anion and cation exchange columns on an Agilent 1100 HPLC. A Polycat A 200 × 4.6 mm, 5 μm, 300-Å column and Polywax LP 100 × 4.6 mm, 5 μm, 1,000-Å column (PolyLC Inc., Columbia, MD) were connected in series for the separation. The injection volume was 100 μL and the column temperature was 35°C. The gradient profile was from 20 mM NH_4_OAc to 1.8 M NH_4_OAc in 9 min and held constant until stopping at 17 min; 10% MeCN was used throughout. Time-based fractions were collected starting at 2.1 min. A 1-min fraction and four 30-sec and three 1-min fractions were collected in order. Samples were dried and resuspended in 100 μL of 3% MeCN, 0.1% formic acid.

WCX-extracted BALF and fractionated whole BALF were separated using the Waters nanoACQUITY UPLC system coupled to a Q-Tof Premier mass spectrometer. The injection volume of 10 μL was trapped using a 180 μm × 20 mm Waters Symmetry C18, 5-μm particle size column using 0.1% formic acid at a flow rate of 5 μL/min for 4 min. The analytical separation was performed using a 75 μm × 100 mm Waters nanoACQUITY UPLC BEH C18 column, 1.7-μm particle size. The column temperature was 35°C. The gradient profile was 3% MeCN for 1 min, 30% MeCN at 101 min, 60% MeCN at 105 min, 80% MeCN at 111 min, and 3% MeCN at 112 min until stopping at 130 min; 0.1% formic acid was maintained throughout. The flow rate was 300 nL/min for the extracted BALF samples and the first fraction of whole BALF. The flow rate was reduced to 250 nL/min because of high back pressure for the remaining fractions.

### Ion trap parameters

Peptides were ionized in positive ion mode. Ion charge control was used with a target of 75,000 counts and a maximum accumulation time of 300 msec. Three precursors were selected based on intensity with an absolute threshold of 1,000 counts. Active exclusion was used and precursors were released after 1 min. The MS/MS fragmentation amplitude was set at 1.2 V.

### QTOF parameters

Peptides were ionized in positive ion mode. Data were collected over the *m/z* 50–1,900 range for 0.8 sec/scan. Scans were performed with the collision cell voltage set at 10 V for low-energy scans and ramped from 20 to 40 V during high-energy scans. [Glu^1^]-fibrinopeptide B was used as an external lock mass for accurate mass calculations (*m/z* 785.8426). A 1-sec lock mass scan was collected every 30 sec.

### Database searches

All searches were performed against an in-house rat database containing the entire rat RefSeq protein database (National Center for Biotechnology Information; www.ncbi.nlm.nih.gov; downloaded 01 March 2006) supplemented with sequences of potentially contaminating proteins, including human keratins, bovine serum albumin (BSA), and trypsin. To control for false positives, random sequences were included in the database. The number, length, and amino acid frequency of the random sequences are equal to those of the downloaded sequences.

Ion trap data were converted to peak lists using DataAnalysis 2.2 (Agilent Technologies). Mascot 2.1 (Matrix Science, Boston, MA) was used for searching with following modifications enabled: fixed carbamidomethyl (C) and variable oxidation (M), oxidation (HW), phospho (STY), sodiated (C-term), and sodiated (DE). A variable imidazole modification was also enabled when appropriate. Under these conditions, the 95% confidence level for an individual peptide match corresponded to Mowse scores ranging from 50 to 54.

The QTOF data were submitted as raw data to Protein Lynx Global Server 2.2 (PLGS; Waters Corp.) and processed using the Protein Expression method. The only modification enabled in PLGS searches was a fixed carbamidomethyl (C). To limit the number of false positives, we considered only protein identifications with confidence levels ≥ 0.99. When multiple isoforms of the same protein were identified that shared numerous identical peptides and could not be distinguished, the confidence levels were summed, and a single protein was reported with multiple accession numbers. Protein identifications determined by direct LC/MS were reported only if they were found in all three technical replicates of at least one condition and had identifications for at least three unique peptides. Despite these rigid criteria, we found that when combining the peak lists from all the LC/MS runs from the fractionated BALF, there was an unacceptably high false positive rate (as determined by the number of random sequence hits). The scoring algorithm used in PLGS appears to overestimate the relevance of numerous, low-quality hits. The effect is pronounced only in large data sets, where presumably the total number of peaks increases the chances that multiple incorrectly identified peptides may be attributed to the same protein in the database. To control for this, we developed a second scoring criterion using the average score per peptide and the average score for the top five highest scoring peptides. Only protein identifications with an average score per peptide > 2.7 or average of the top five peptides > 10 were considered to be high-quality hits. These values were set at a level that limited the false positive rate to < 5% in single replicates and allowed no detectable false positives when the replicate filter was used.

## Results

**Figure 1:**
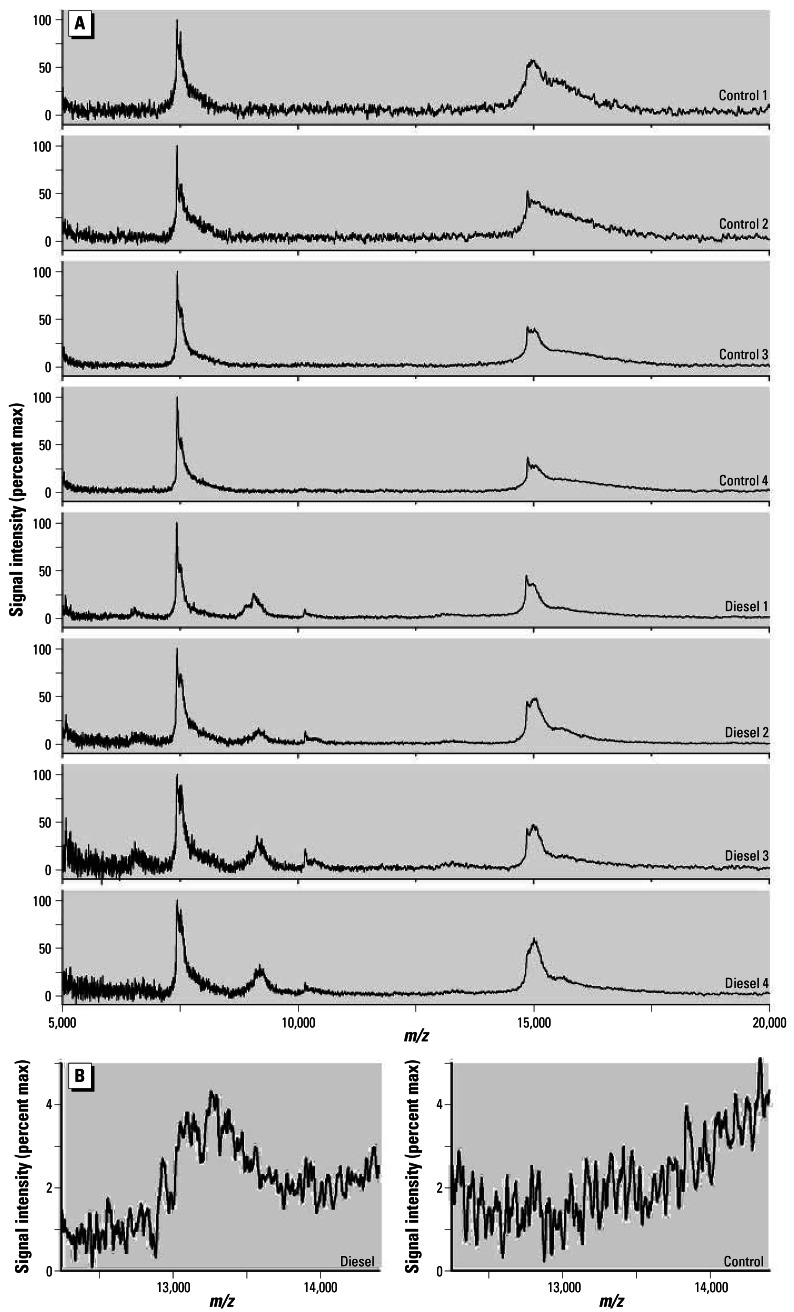
(*A*) SELDI-TOF mass spectra of BALF obtained at 24 hr post-treatment from four control rats (PBS instilled) and four rats exposed intratracheally to 50 mg DEP/kg body weight and (*B*) a zoomed view of two spectra generated by averaging the data from the four control or four exposed replicates. max, maximum. Peaks at 9,100 and 10,100 are detected only in the spectra from the exposed samples. The peak near 13,100 is also present only in exposed samples but is undoubtedly only above baseline when multiple spectra are averaged.

Figure 1A shows the SELDI-TOF spectra of BALF using a WCX2 ProteinChip obtained from four control and four DEP-exposed animals at 50 mg/kg body weight. When compared with the mass spectra from the control animals, the spectra from the DEP-exposed animals show two additional peaks with *m/z* values of approximately 9,100 and 10,100. These peaks appear in samples from all the exposed doses at 24 hr but are not seen in day 7 and day 30 samples (data not shown). This finding indicates that these peaks represent an acute response to DEP exposure that resolves in a few days.

**Figure 2:**
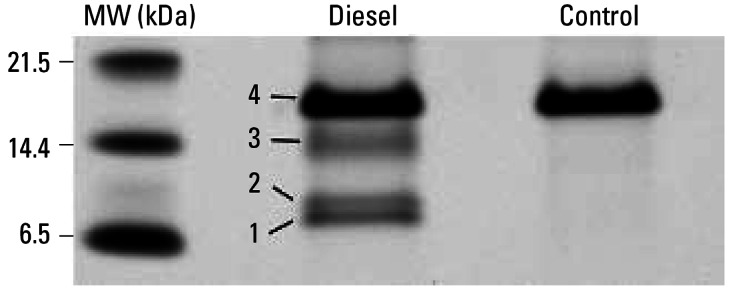
Gel electrophoresis of extracted BALF obtained at 24 hr posttreatment from a control rat (PBS instilled) and a rat exposed intratracheally to 50 mg of DEP/kg body weight. The four numbered bands in the diesel lane were excised and analyzed by LC/MS.

To identify these proteins, we fractionated BALF from rats 24 hr postexposure by SPE using a WCX resin with subsequent denaturing polyacrylamide gel electrophoresis (SDS-PAGE). Four predominant bands are observed in exposed samples in the low-molecular-weight region of the gel where the proteins corresponding to the peaks of interest from the SELDI-TOF data would be expected to migrate (Figure 2). These gel bands were excised; the protein contained in them was digested, eluted, and analyzed by LC/MS technology using an ion trap mass spectrometer. The uppermost band (Figure 2, band 4) is present at approximately the same concentration in both exposed and unexposed samples and was identified as lysozyme. The two lowest bands (Figure 2, bands 1 and 2) likely correspond to the SELDI-TOF peaks with *m/z* 9,100 and 10,100 and were identified by database searches as anaphylatoxin C3a and calgranulin A. We could not identify the protein from the middle band (Figure 2, band 3, *M_r_* = 10–15 kDa) by analysis of the excised gel slice. A small peak corresponding to its relative molecular weight is detectable in the SELDI-TOF data with an *m/z* just above 13,000. However, it is only distinctly above noise level when multiple spectra are averaged (Figure 1B).

Two predominant peptides (Table 1) from the SDS-PAGE-excised bands formed the basis for the identification of anaphylatoxin C3a, which is a proteolytically processed product of complement C3. Two peptides present in complement C3 but not in anaphylatoxin C3 were identified, suggesting that complement C3 or partially cleaved C3 might be present. However, we believe that the identification of these peptides as C3 sequences is artifactual because they were the two lowest scoring peptide matches in the search (scores = 3 and 6), their scores were below statistical significance, and they were only found in imidazole-labeled samples, even though neither peptide has this modification. We conclude that the presence of naturally processed anaphylatoxin C3a is the most likely explanation for the presence of complement C3 peptides.

SPE-fractionated BALF was also analyzed directly by LC/MS (no SDS-PAGE) using a QTOF and the Waters Protein Expression method, and the presence of calgranulin-A was confirmed (Table 2). Complement C3 or a C3 isoform (XP_579384), 98% identical to C3 overall and 100% identity in the anaphylatoxin C3a region) was also identified, but since proteins are digested with trypsin before LC/MS analysis, the full-length and processed proteins are indistinguishable. Additionally, this technique provided a possible protein identification for the previously unidentified band (Figure 2, band 3) and the small SELDI-TOF peak at 13,000 (Figure 1B), calgranulin B.

A total of 65 proteins were identified by performing LC/MS analysis of whole BALF and WCX-extracted BALF (Table 3) on a QTOF using the Waters protein expression method. Each reported protein was identified in all three technical replicates of at least one of the conditions. We compared the lists of confirmed proteins and unfiltered search results to identify possible missed or lower scoring identifications. The quality of the identification of each protein in each condition was assigned in one of four ranks: *a*) high-quality identifications in all three replicates, *b*) a high-quality identification in at least one replicate, *c*) a low-quality identification in at least one replicate, and *d*) not identified. The majority of the proteins (41) were seen in both control and exposed samples, whereas 20 were identified only in diesel-exposed samples and 4 were identified only in control samples.

**Figure 3:**
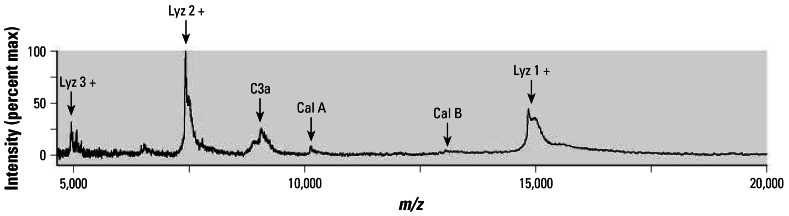
Protein identification for SELDI-TOF peaks. max, maximum. The predominant peaks identified by MS/MS or MS^E^ in BALF obtained at 24 hr postexposure to DEP are labeled with the corresponding protein: anaphylatoxin C3a (C3a), calgranulin A (Cal A), calgranulin B (Cal B), or lysozyme (Lyz). The three different charge states observed for lysozyme are indicated.

The predominant peaks found in the SELDI-TOF spectra have all been identified (Figure 3). Lysozyme (Lyz) is quite abundant in these samples and appears as singly, doubly, and triply charged peaks (*m/z* 15,000, 7,500, and 5,000, respectively). Two of these (Figure 3; Lyz 1+ and Lyz 2+) are the largest peaks in all the samples and do not change as a result of exposure. Two readily observable peaks, seen only in spectra from the diesel-exposed, 24-hr samples, represent anaphylatoxin C3a and calgranulin A, and a third peak, whose signal is only slightly above noise, is also present only in spectra from exposed, 24-hr samples and was identified as calgranulin B.

## Discussion

Diesel exhaust particles are generated by heavy-duty diesel engines used in many industries and motor vehicles used in public transportation. They are respirable particles with an average diameter of 250 nm and contain several mutagenic and carcinogenic hydrocarbons (Arlt et al. 2003). Epidemiologic and experimental animal studies have shown an increased risk of respiratory and cardiovascular morbidity and mortality associated with exposure to DEPs (Rai et al. 2002; Salvi 2001; Steerenberg et al. 1998). They also cause adverse reactions in the lungs (Diaz-Sanchez et al. 1994) and other tissues (Yoshino and Sagai 1999). DEP exposure induces production of cytokines in AMs (Yang et al. 1997, 1999), in lung epithelial cells, and in lung tissue (Bayram et al. 1998; Saber et al. 2006; Steerenberg et al. 1998). Within 24 hr after exposure to DEPs, quantifiable changes in cytokines, an influx of inflammatory cells and proteins, and up-regulation of gene expression of inflammatory mediators are observable in BALF from exposed rats (Rao et al. 2005).

The aim of this study was to characterize the changes in protein profiles in the BALF of rats following DEP exposure using newly available proteomic technologies. This was accomplished using two complementary technologies, SELDI-TOF and LC/MS. The spectra obtained using a Ciphergen ProteinChip System contain two readily observable peaks and a third weak peak that are specific to BALF samples taken from rats 24 hr after exposure to DEPs. Subsequent analysis using LC/MS indicates that the proteins producing these peaks are calgranulin A, calgranulin B, and anaphylatoxin C3a.

An additional 62 proteins present in BALF were also identified through the utilization of Waters’s LC/MS protein expression technology. Twenty (20) proteins, most of which are lung damage and inflammation specific, were repeatedly identified only in the exposed sample. Presumably, they are more abundant in this sample, as there is a strong bias toward identification of the proteins at the highest concentrations using LC/MS. Four proteins were identified in the control sample but not in the exposed one. The abundance of these proteins may have been reduced in BALF from exposed animals. However, the presence of these proteins may also simply have been masked in the exposed sample by the higher amount of total protein in it.

There is a large difference in total protein levels between the two samples, with the higher protein concentration in the exposed sample likely a result of plasma extravasation. Consistent with this view, many of the plasma-derived proteins identified in both samples do indeed change in abundance [for example, albumin (Rao et al. 2005 and unpublished data)], but additional work will be required to provide accurate quantification. A quantitative comparison using the Protein Expression method was confounded by ion-suppression effects and challenges in normalization resulting from the large difference in total protein concentrations. Because we prefer to interpret the data conservatively, we have not reported this “quantitative” data.

A side note to the LC/MS analysis is that different sets of proteins were identified in the SPE samples and in the whole BALF. The largest hindrances to protein identification by mass spectrometry are sample complexity and dynamic range. The WCX extraction addresses both of these by reducing the concentration of the most abundant protein (albumin) and reducing the total number of proteins present. This is why proteins that are “hidden” in whole BALF can be identified in SPE samples.

Based on the proteins identified, the major observed effect of DEP exposure appears to be an inflammatory response. Anaphylatoxin C3a, a component of the complement system, is a well-known mediator of inflammation [see Ember and Hugli (1997) for a review], and calgranulin A is a part of a heterodimer with calgranulin B (also known as MRP-8 and MRP-14, respectively) that make up calprotectin. Calprotectin is currently used as a biomarker of inflammation for several human diseases. It is primarily expressed in PMNs and is estimated to account for 30–60% of their cytosolic and 5% of their total proteins. Furthermore, the level of calprotectin at sites of inflammation is known to correlate with the number of localized PMNs [reviewed in Striz and Trebichavsky (2004); Yui et al. 2003], and we have previously shown that exposure to DEPs causes an increase in the PMN content of BALF at 24 hr (Rao et al. 2005). The presence of an inflammatory response is further supported by the qualitative analysis of the proteins identified by LC/MS.

Many of the proteins that we have observed in both the exposed and unexposed sample are highly abundant in the plasma and are present as a result of plasma extravasation. However, DEP-exposed samples show a pronounced increase in the amount and number of proteins observed, which appears to be caused by damage at the air–blood barrier that is a result of DEP exposure (Rao et al. 2005). Without analysis of the plasma, it is not possible to discriminate changes in concentration of plasma-derived proteins that are present because of extravasation from those that are specific to the inflammatory responses.

On the technical side, it is worth noting that the discovery of anaphylatoxin C3a in the SELDI-TOF data demonstrates an advantage to a top-down proteomics approach. LC/MS analysis of digested proteins is unable to distinguish processed and unprocessed complement C3. The SDS-PAGE analysis confirmed the SELDI-TOF data but required significantly more effort to acquire the data. Protein modifications, such as the cleavage of complement C3, can play major biological roles and are important to characterize. However, it must be noted that the top-down approach used in this work is not a global analysis, as only proteins that bind with high affinity to a weak cation exchange chip were retained. The WCX2 chip was chosen because of its low affinity for serum albumin, which is abundant in BALF; in the future the analysis could be extended by using chips supporting other surface chemistries. Another caveat was that this approach only detected large acute changes and was unable to discern difference at later time points. Since it is known that the lungs have not returned to normal (Rao et al. 2005), it is likely that the method was not sensitive enough or does not have sufficient dynamic range to identify the lower abundance proteins that are perturbed at 7 or 30 days postexposure. In the current state of proteomic technology, a combination of complementary strategies is required to maximize proteome coverage.

The protein expression method used in this study provides more complete coverage than the SELDI-TOF and was chosen over a traditional LC-MS/MS approach because it is more suited for comparative analyses. This method of fragmentation provides more reproducible coverage because all the ions are fragmented each run, and the identified peptides are not limited by which precursor ions are select for tandem MS. Furthermore, since only one fragmented ion scan is performed for each survey scan, there is far more quantitative data for the parent ions. This being said, two noteworthy shortcomings were identified with this approach. The scoring algorithm used for database searches needs to be optimized. We addressed the issue by removing proteins that were identified as the result of multiple low scoring peptide hits, but ideally such filtering should be handled in the initial search. There is no easy way to normalize or to account for ion suppression resulting from samples with large difference in protein abundance, such as the unexposed and exposed BALF samples.

In summary, we demonstrate that it is possible to detect markers of inflammation after diesel exhaust particulate exposure in the BALF using the Ciphergen ProteinChip System. Additional mass spectrometric investigation using liquid chromatography coupled to mass spectrometry (LC/MS) was used to identify the predominant peaks present in the SELDI-TOF spectra and also provided an additional list of proteins that change in response to exposure. Further studies are required to see if these markers are detectable in serum samples from animals or humans exposed to diesel exhaust.

## Figures and Tables

**Table 1:** Gel band identification of proteins corresponding to SELDI-TOF peaks.^a^

Gel band	High score^b^	No. of spectra	Processing^c^	Sequence	Modifications^d^	Protein^e^
1	6	1	IMID	ILLQGTPVAQMAEDAVDGERLK		C3
1	56	2	IMID	LITQGESCLK	IMID	C3a
1	34	1		AFMDCCNYITK	ox-Met	C3a
1	34	1		LITQGESCLK		C3a
1	87	6		MVTTECPQFVQNK	ox-Met	cal A
1	66	1		MVTTECPQFVQNK		cal A
2	3	1	IMID	FGLEKR		C3
2	11	1	IMID	ARLITQGESCLK	IMID	C3a
2	79	6	IMID	LITQGESCLK	IMID	C3a
2	64	3		AFMDCCNYITK	ox-Met	C3a
2	59	6		LITQGESCLK		C3a
2	64	1		MVTTECPQFVQNK	ox-Met	cal A

a Protein bands excised from an SDS-PAGE gel (Figure 2) from BALF sample taken 24 hr postexposure were digested and analyzed by LC-MS/MS on an ion trap mass spectrometer. Peptide identifications were determined using Mascot.

b Highest score for an individual spectrum from the Mascot search for the indicated peptide.

c An aliquot of each sample was labeled with imidazole before MS analysis to enhance ionization. This indicates whether the identification was in a modified or unmodified sample.

d Modifications identified by Mascot search (IMID–imidazole or ox-Met–oxidated methionine).

e Protein identifications are complement C3, anaphylatoxin C3a, or calgranulin A (cal A).

**Table 2:** LC/MS identification of proteins corresponding to SELDI-TOF peaks.^a^

Replicate	Condition	Peak (*m/z*)	Score	Unique peptides	Protein
1	Exposed	9,100	58.9	3	Calgranulin A
2	Exposed	9,100	49.0	4	Calgranulin A
3	Exposed	9,100	51.3	4	Calgranulin A
1	Exposed	10,100	51.8	25	Complement C3
1	Exposed	10,100	60.5	33	XP_579384
2	Exposed	10,100	39.8	26	Complement C3
2	Exposed	10,100	38.7	26	XP_579384
3	Exposed	10,100	107.5	29	XP_579384
1	Exposed	13,200	43.4	3	Calgranulin B
2	Exposed	13,200	53.0	7	Calgranulin B
3	Exposed	13,200	30.8	4	Calgranulin B
1	Exposed	5,000^b^, 7,500,15,000	107.0	7	Lysozyme
2	Exposed	5,000^b^, 7,500,15,000	61.3	6	Lysozyme
3	Exposed	5,000^b^, 7,500,15,000	120.8	7	Lysozyme
1	Unexposed	5,000^b^, 7,500,15,000	56.4	5	Lysozyme
2	Unexposed	5,000^b^, 7,500,15,000	58.0	5	Lysozyme
3	Unexposed	5,000^b^, 7,500,15,000	92.9	8	Lysozyme

a Proteins were extracted using a weak cation exchange resin from BALF obtained at 24 hr posttreatment from a control rat (PBS instilled) and a rat exposed intratracheally to 50 mg of DEP/kg body weight. Tryptic digests of the proteins were analyzed on a QTOF using the Protein Expression method and identified using PLGS.

b The *m/z* of 5,000 and 7,500 corresponds to triply- and doubly-charged lysozymes.

**Table 3:** LC/MS identification of proteins in BALF from rats.^a^

GenInfo no.^b^	Description	Gene symbol	Origin^c^	WCX_D^d^	Diesel	WCX_C	Control	Found^e^
27229290	afamin	*Afm*	Plasma	—	+++	—	+++	Both
19705431	albumin	*Alb*	Plasma	+++	+++	+	+++	Both
83816939	alpha 1 inhibitor III	*Mug1*						
62648373	alpha 1 inhibitor III	*Mug1*	Plasma	—	+++	—	+++	Both
62647940	alpha 1 inhibitor III	*Mug1*						
12831225	alpha 1 inhibitor III	*Mug1*						
6978477	alpha 2 HS glycoprotein	*Ahsg*	Plasma	++	+++	+	+++	Both
34867677	alpha-1-antichymotrypsin	*Serpina3m*	Plasma	—	+++	—	+++	Both
51036655	alpha-1-antitrypsin	*Serpina1*	Plasma	—	+++	—	+++	Both
58865630	antithrombin-III	*Serpinc1*	Plasma	—	++	—	+++	Both
6978515	apolipoprotein A I	*Apoa1*	Plasma	—	+++	—	+++	Both
57528174	apolipoprotein H	*Apoh*	Plasma	+++	+++	—	+++	Both
57529187	carboxylesterase, esterase 2	*Es2*	Leukocytes	—	+++	—	+++	Both
6978695	ceruloplasmin	*Cp*	Plasma	—	+++	—	+++	Both
61657901	chitinase 3 like 1	*Chi3l1*	Leukocytes	++	+	—	+++	Both
47059181	complement B factor	*Cfb*	Plasma	+++	+++	+++	+++	Both
47575877	complement component 2	*C2*	Plasma	+	+++	—	+	Both
8393024	complement component 3	*C3*	Plasma	+++	+++	+++	+++	Both
62718645	complement component 3	*C3*						
29789265	complement component 4a	*C4a*	Plasma	+++	—	—	+++	Both
54234046	cystatin C	*Cst3*	Leukocytes	—	+++	—	++	Both
17865327	fetuin beta	*Fetub*	Plasma	—	+++	—	+++	Both
29789106	fibrinogen beta polypeptide	*Fgb*	Plasma	+++	+	—	+++	Both
51854227	gelsolin	*Gsn*	Plasma	+++	+++	+	—	Both
6978879	group specific component	*Gc*	Plasma	—	+++	—	+++	Both
60097941	haptoglobin	*Hp*	Plasma	++	+++	+	+++	Both
17985949	hemoglobin beta chain	*Hbb*	Blood	++	+++	—	+++	Both
16758014	hemopexin	*Hpx*	Plasma	+++	+++	+	+++	Both
62651518	immunoglobulin heavy chain like		Plasma	+	+++	—	+	Both
9506819	inter alpha inhibitor H4 heavy chain	*Itih4*	Plasma	+	+	—	+++	Both
80861401	kininogen 1	*Kng1*	Plasma	—	+++	—	+++	Both
40254796	lysozyme	*Lyz*	Lung	+++	+++	+++	+++	Both
25282393	mast cell peptidase 2	*mcpt2*	Leukocytes	—	+++	+	—	Both
27465565	Niemann Pick type C2	*Npc2*	Leukocytes	—	+++	+	++	Both
62638541	plasminogen	*Plg*	Plasma	++	+++	—	+++	Both
21955142	pregnancy zone protein	*Pzp*	Plasma	+++	+++	—	+++	Both
6981694	secretoglobin family 1A	*Scgb1a1*	Lung	—	+++	+	++	Both
18266692	selenium binding protein 2	*Selenbp1*	Lung	—	+++	—	++	Both
32563565	serine protease inhibitor 2a	*Spin2a*	Plasma	—	+++	—	+++	Both
6981576	serine protease inhibitor 2b	*Spin2b*	Plasma	—	+++	—	+++	Both
13928716	serine protease inhibitor 2c	*Serpina3n*	Plasma	++	+++	—	+++	Both
20301980	surfactant associated protein B	*Sftpb*	Lung	—	+++	—	+++	Both
7949133	surfactant associated protein D	*Sftpd*	Lung	+++	+++	+++	+++	Both
62654137	transferrin	*Tf*	Plasma	+++	+++	+++	+++	Both
61556986	transferrin	*Tf*						
62654202	transferrin like		Plasma	—	+++	—	++	Both
16758048	advanced glycosylation end product-specific receptor	*Ager*	Lung	—	+++	—	—	DEP
6978501	annexin A1	*Anxa1*	Lung	—	+++	—	—	DEP
6978505	annexin A5	*Anxa5*	Lung	—	+++	—	—	DEP
34861019	calcium activated chloride channel	*Clca3*	Lung	—	+++	—	—	DEP
16758672	calgranulin A	*S100a8*	Leukocytes	+++	—	—	—	DEP
16758364	calgranulin B	*S100a9*	Leukocytes	+++	—	—	—	DEP
62078741	coagulation factor XII	*F12*	Plasma	+++	—	—	—	DEP
77861917	complement component factor H	*Cfh*	Plasma	+++	++	—	—	DEP
25742583	defensin beta 3	*Defb3*	Lung	—	+++	—	—	DEP
62643670	fibrinogen alpha polypeptide	*Fga*	Plasma	+++	—	—	—	DEP
56797757	fibrinogen alpha polypeptide	*Fga*						
62657833	histidine rich glycoprotein	*Hrg*	Plasma	+++	—	—	—	DEP
19173806	histidine rich glycoprotein	*Hrg*						
62079255	immunoglobulin heavy chain like		Plasma	—	+++	—	—	DEP
62660301	immunoglobulin joining chain	*Igj*	Plasma	—	+++	—	—	DEP
25282405	palate lung and nasal epithelium carcinoma associated protein	*Plunc*	Lung	—	+++	—	—	DEP
16758348	peroxiredoxin 6	*Prdx6*	Lung	—	+++	—	—	DEP
27151742	polymeric immunoglobulin receptor	*Pigr*	Lung	—	+++	—	—	DEP
62660728	SEC14 like 3	*Sec14l3*	Lung	—	+++	—	—	DEP
8394337	surfactant pulmonary-associated protein A1	*Sftpa1*	Lung	—	+++	—	—	DEP
6981684	transthyretin	*Ttr*	Plasma	+++	—	—	—	DEP
27465549	WAP four disulfide core domain 2	*Wfdc2*	Lung	—	+++	—	—	DEP
19705570	angiotensinogen	*Agt*	Plasma	—	—	—	+++	Cont
8393197	C reactive protein	*Crp*	Plasma	—	—	—	+++	Cont
62658037	carboxypeptidase N regulatory subunit	*Cpn2*	Plasma	—	—	—	+++	Cont
40018558	complement component 1 inhibitor	*Serping1*	Plasma	—	—	—	+++	Cont

a Tryptic digests of proteins from BALF obtained at 24 hr posttreatment from a control rat (PBS instilled) and a rat exposed intratracheally to 50 mg of DEP/kg body weight were analyzed on a QTOF using the Protein Expression method and identified using PLGS.

b The GI number is a unique GenInfo identifier for the protein sequence in the NCBI’s GenBank database (www.ncbi.nlm.nih.gov).

c Probable origins of proteins.

d The quality of identification for each protein in each sample was categorized into one of four levels: high-scoring identifications in all three technical replicates (+++), a high-scoring identification in at least one replicate (++), a low-scoring identification in at least one replicate (+), or no identification (—). Columns represent SPE extracted diesel or control samples (WCX_D or WCX_C, respectively) or whole BALF from diesel or control samples.

e Indicates whether proteins were identified in exposed, unexposed, or both samples (DEP, Cont, or Both, respectively).
